# Outer Membrane Vesicles Derived from Yak Isolates of *Pasteurella multocida* Exhibit Promising Vaccine Potential

**DOI:** 10.3390/ani16081264

**Published:** 2026-04-20

**Authors:** Chao Jin, Kewei Li, Haofang Yuan, Xiaohu Zhang, Muhammad Farhan Rahim, Yaozhong Lu, Siyang Mu, Shan Wu, Hang Su, Xiaoqiang He, Zhun Yi, Hongbin Yin, Jiakui Li

**Affiliations:** 1 College of Veterinary Medicine, Huazhong Agricultural University, Wuhan 430070, China; 2024302010142@webmail.hzau.edu.cn (C.J.); lzx591387332@163.com (K.L.); yhf9910@163.com (H.Y.); farhan092@webmail.hzau.edu.cn (M.F.R.); luyaozhong@webmail.hzau.edu.cn (Y.L.); msy1011@webmail.hzau.edu.cn (S.M.); wushan53@webmail.hzau.edu.cn (S.W.);; 2 Abz Academy of Agricultural Sciences, Aba 624000, China; 18030443900@163.com (X.H.); 18328817870@163.com (Z.Y.); 3 Yunnan Province Animal Disease Prevention and Control Center, Kunming 650201, China; 4 Hubei Jiangxia Laboratory, Wuhan 430200, China

**Keywords:** *Pasteurella multocida*, outer membrane vesicles, vaccine candidate, yaks, immunogenicity, protective efficacy, macrophage activation

## Abstract

*Pasteurella multocida* (*P. multocida*) is a significant pathogenic bacterium that causes serious disease and death in the yaks of the Tibetan Plateau, and the existing inactivated vaccines are limited by low protection and reactogenicity. Outer membrane vesicles (OMVs) derived from a yak-origin serogroup B *P. multocida* isolate were evaluated as a potential vaccine candidate in the present study. The purified OMVs were characterized by transmission electron microscopy and nanoparticle tracking analysis, which demonstrated the presence of typical bilayer vesicles ranging from 20 to 300 nm in diameter. Proteomic profiling revealed 1213 proteins, with many of them being immunologically relevant outer membrane-associated proteins like OmpA, OmpH, Omp16, OmpW, TbpA and PlpP. The functional enrichment analysis showed that these proteins were linked to translation, membrane structure, transport, metabolism, and pathways of adaptation of bacteria. In vitro OMVs were effectively taken up by RAW264.7 macrophages and stimulated robust expression of inflammatory mediators, such as TNF-α, IL-1β, IL-6, iNOS and IL-10, which is indicative of strong innate immunostimulatory capacity. OMV immunization induced significant antigen specific humoral responses in mice and yaks in vivo. In mice, intramuscular immunization was effective in giving full protection against *P. multocida* challenge but not intranasal immunization. Histopathology also indicated less tissue damage in vaccinated animals, especially in the lung and liver. These findings, taken together, prove that yak-derived *P. multocida* OMVs have high immunogenicity and protection capabilities, which show their potential as a next-generation vaccine platform to tackle *P. multocida* infection.

## Simple Summary

Outer membrane vesicles derived from yak-origin *Pasteurella multocida* exhibited notable immunogenicity and protective efficacy, indicating their promise as a next-generation vaccine candidate against hemorrhagic septicemia in yaks. These vesicles displayed characteristic nanoscale morphology, contained multiple antigenic outer membrane-associated proteins, and efficiently activated macrophage-mediated innate immune responses. Immunization induced strong antigen-specific antibody responses in both murine and yak models, and intramuscular administration conferred complete protection against homologous challenge in mice while markedly reducing pathological lesions. Overall, the study provides important evidence that OMV-based vaccination may represent an effective and translational strategy for the prevention of *P. multocida* infection in livestock.

## 1. Introduction

*Pasteurella multocida* (*P. multocida*) was initially isolated in 1881 by the French microbiologist Pasteur [1]. *P. multocida* is morphologically a small, non-motile, non-flagellated, Gram-negative coccobacillus [2]. It is capable of infecting numerous livestock, wild animals and human beings [3]. Various serotypes of *P. multocida* infect different host species and are classified on the basis of their capsular and lipopolysaccharide (LPS) antigens. Among these, avian cholera isolates most frequently belong to serotypes A:1, A:3, and A:4 [4]. The serotype B:2 and E:2 are normally related to bovine hemorrhagic septicemia [5]. Serotype A:3 is most commonly associated with bovine respiratory disease and is more prevalent in calves, especially following transportation stress, whereas serotype B is linked to bovine hemorrhagic septicemia, which is characterized by high mortality after onset [6,7,8]. Serotype A or D tends to cause progressive atrophic rhinitis in pigs [9]. *P. multocida* infection in rabbits can be commonly associated with serotypes A:3, A:4, and A:12 [10]. The infection of humans is uncommon and is normally caused by animal bites or scratches, although older and immunocompromised people are more prone to infection [11].

Vaccination is the most effective and economical method to protect animals from *P. multocida* or to reduce symptoms to the mildest extent possible [12]. However, the most widely used inactivated vaccines have drawbacks such as large injection doses, low cross-protection, and noticeable injection site inflammation [13,14,15].

Outer membrane vesicles (OMVs) are nanoscale lipid bilayer structures released by bacteria into the extracellular environment, typically measuring 20 to 400 nm in diameter [16]. These vesicles are predominantly derived from the bacterial outer membrane and carry a range of membrane-associated components, such as LPS, proteins, and phospholipids [16]. Because of their critical roles in bacterial survival, virulence, and interactions with the host and external environment, OMVs have been extensively investigated in recent years [17]. The domestic yak (*Bos grunniens*) is an iconic and essential livestock species endemic to the high-altitude regions of the Qinghai–Tibet Plateau. Due to the extreme environmental conditions and the challenges of traditional extensive husbandry, yaks are highly susceptible to bovine hemorrhagic septicemia, which remains a leading cause of mortality. Therefore, developing a novel and effective vaccine against *P. multocida* is crucial to reducing mortality rates and mitigating the risk of disease transmission in these unique pastoral systems. Notably, while outer membrane proteins (OMPs) of *P. multocida* and OMVs derived from porcine, avian, and bovine strains have been extensively investigated, no systematic study has been reported on OMVs from yak-pathogenic *P. multocida* serogroup B strains, which are the dominant epidemic strains causing fatal hemorrhagic septicemia in yaks in the Qinghai–Tibet Plateau. Furthermore, almost all existing *P. multocida* OMV studies were limited to mouse models, and their immunogenicity and protective efficacy in the natural host (yak) remain completely unknown. The present study mainly aims to isolate and characterize OMVs derived from yak-specific *P. multocida* for immunogenicity evaluation. Here, we improved the limitations of current inactivated vaccines by exploring OMVs as a new vaccine candidate against *P. multocida* infections in yaks by characterizing the OMVs based on their morphology, size distribution, and protein composition.

## 2. Material and Methods

### 2.1. Preparation of Pasteurella multocida Outer Membrane Vesicles

A *Pasteurella multocida* strain was isolated from infected yaks in Tibet, China, by our group at Huazhong Agricultural University, identified as serogroup B, and stored at −80 °C until use. To recover, the frozen stock was thawed slowly in ice, streaked onto tryptic soy agar (TSA) with the addition of 5% defibrinated sheep blood, and incubated at 37 °C 24 h. One colony was then inoculated in 5 mL of tryptic soy broth (TSB) and allowed to grow at 37 °C, 12 h. The seed culture was then inoculated into 2 L of TSB and left to incubate at 37 °C for a further 12 h to expand the bacteria in large numbers. After incubation, bacterial cells were centrifuged at 8000× *g* within 30 min at 4 °C. The cell-free supernatant was subsequently concentrated by 100 kDa tangential flow filtration (TFF, Sartorius, Goettingen, Germany). The resulting concentrate was then processed for OMV isolation and purification by Gemexo Biotech (Wuhan, China). The purified OMVs were stored at −80 °C until use.

### 2.2. Transmission Electron Microscopy (TEM) and Nanoparticle Tracking Analysis (NTA)

The OMVs were pooled into fractions and subjected to transmission electron microscopy (TEM) and nanoparticle tracking analysis (NTA). For TEM, the OMV samples were first fixed on ice in a solution containing 2% paraformaldehyde and 2.5% glutaraldehyde. After fixation, the samples were washed with phosphate-buffered saline (PBS) and post-fixed in 1% osmium tetroxide for 1 h at room temperature to preserve vesicular morphology and ultrastructural integrity. The samples were then dehydrated through a graded ethanol series (30%, 50%, 70%, 90%, and 100%) and subsequently embedded in epoxy resin. An ultramicrotome was used to make ultrathin sections (70–90 nm) which were mounted on copper grids with formvar coating. To improve the contrast of the images, 2% uranyl acetate staining (15 min) and lead citrate (5 min) were applied to the sections [18]. Grids were analyzed under a JEOL-1010 (JEOL, Tokyo, Japan) transmission electron microscope at an accelerating voltage of 80 kV, and digitally recorded images were taken. NTA was also used to analyze the size distribution and particle concentration of OMVs. Before the analysis, the OMV samples were diluted in PBS to obtain the best particle concentration to measure. NTA was done under a NanoSight NS300 (Malvern Panalytical, Worcestershire, UK) set-up with a 488 nm laser. The concentration and size of the particles were determined using the Brownian motion of vesicles suspended in solution [19].

### 2.3. RAW264.7 Macrophages Culture

BALB/c monocyte-macrophage leukemia cells (CCTCC, TCM13) were cultured in Dulbecco’s Modified Eagle’s Medium (DMEM; Gibco, 10566016) with 10% (*v*/*v*) fetal bovine serum (FBS; Gibco, A5256701) at 37 °C in a humid atmosphere containing 5% CO_2_.

### 2.4. Cytotoxicity Assay and Analysis of Cytokine Transcription Levels

To ascertain the non-cytotoxic concentration of the *P. multocida* OMVs, RAW264.7 cells were seeded in 96-well plates at a concentration of 1 × 10^4^ cells per well and incubated overnight to allow the cells to settle. The OMVs were then added to the cells at the final concentrations of 0.1, 0.5, 1, 5, 10, 25, 50, 75 and 100 μg/mL after 24 h; the control group cells were treated with the same volume of PBS. CCK-8 reagent (100 μL) was put on the stems of each well, and the plates were further incubated at a dark location (1 h). A microplate reader was then used to measure the absorbance at 450 nm. RAW264.7 cells were cultured into six wells at 1 × 10^6^ cell density and allowed to adhere, and then the effect of OMVs on the transcription of cytokines was assessed. After that, OMVs 0.5 μg/mL, 1 μg/mL LPS or the same volume of PBS were added to the cells and left alone for 5 h. The entire RNA was isolated, and the expressions of TNF-α, IL-1β, IL-6, IL-10 and iNOS mRNAs were identified.

### 2.5. Cellular Uptake of Outer Membrane Vesicles

Cells were grown on sterile coverslips in 12-well plates at a concentration of 2 × 10^5^ cells/well and incubated overnight to allow the cells to attach. Labeled with BeyoExo™ Exosome Labeling and Tracking Kit containing PKH26 (C3637, Beyotime, Shanghai, China), OMVs were then added at 30 μg/well, and the cells were incubated for 1, 2 or 4 h. After incubation, paraformaldehyde was used to fix the cells, and Phalloidin (Alexa Fluor 488, ThermoFisher, Waltham, MA, USA) and DAPI (1 mg/mL, Solarbio, Beijing, China) were subsequently used to stain the F-actin and the nuclei, respectively. A laser scanning confocal microscope (AXR NSPARC, Nikon, Tokyo, Japan) was then used to examine the coverslips.

### 2.6. Proteomic Characterization of OMVs

The construction of the proteomic library and protein quality identification were performed by Gemexo Biotech (Wuhan, China). OMV samples (1 μg protein/sample) were reduced with 5 mM dithiothreitol at 37 °C over 1 h and alkylated with 10 mM iodoacetamide at room temperature in the dark. The samples were diluted four-fold in 25 mM ammonium bicarbonate and digested overnight at 37 °C with trypsin at a protein–enzyme ratio of 50:1 (*v*/*v*). The action of enzymatic digestion was stopped by adding formic acid to bring the pH to an approximate value below 3. The desaltation of peptides was performed with C18 columns, and it was pooled with 0.1 formic acid, eluted with 70% acetone, dried under a lyophilizer and analyzed under the HPLC-MS/MS system. A RIGOL L-3000 HPLC (RIGOL, Suzhou, China) system took part in a peptide separation procedure whereas a Q Exactive HF-X (ThermoFisher, Waltham, MA, USA) mass spectrometer with a Nanospray Flex^TM^ (ThermoFisher, Waltham, MA, USA) electrospray ionization source was used in mass spectrometric analysis. The acquisition was data-dependent between 350 and 1500 m/z. MS scans of full MS were obtained with a resolution of 120,000, and the top 40 most abundant precursor ions were fragmented using HCD. The resolution of the MS/MS spectra was 15,000. The target of the AGC was established to 3 × 10^6^ and 5 × 10^4^ of MS and MS/MS, respectively, and the maximum injection time was 80 and 45 ms, respectively. The normalized collision energy was adjusted to 27%. Protein identification was done with the Proteome Discoverer v2.4 against the UniProt *P. multocida* reference database (strain Pm70; UP000000809). Trypsin/P was indicated as the digestion enzyme and had up to two missing cleavages. The fixed and variable modifications were established as carbamidomethylation of cysteine and oxidation and acetylation of methionine and protein N-terminal, respectively. The mass tolerances were set to 15 ppm when using precursor ions and 0.02 Da when using fragment ions. Identified proteins were functionally annotated with UniProt-GOA database and InterProScan, and proteins were grouped into Gene Ontology (GO) classifications, such as Biological Process, Molecular Function, and Cellular Component. Kyoto Encyclopedia of Genes and Genomes (KEGG) pathway enrichment KEGG Mapper and KAAS were used to perform KEGG pathway enrichment.

### 2.7. Mouse Immunization and Bacterial Challenge

Immunization and challenge studies were conducted in six-week-old female BALB/c mice. All animal experiments were performed in accordance with the guidelines of the Animal Welfare and Ethics Committee of Huazhong Agricultural University and were approved by that committee. Mice were immunized either intramuscularly with 50 µg OMVs in 100 µL PBS or intranasally with 25 µg OMVs in 10 µL PBS, whereas control mice received 100 µL PBS alone. The primary and booster immunization took place on days 0 and 14, respectively. Blood samples were taken before immunization, 1 and 2 weeks after the initial immunization, and 2 weeks after the booster dose. The serum was collected and frozen at −80 °C to be analyzed later. Mice were challenged by intraperitoneal inoculation with a target dose of 101 CFU of a *P. multocida* isolate isolated in yaks 4 weeks after the first immunization. Survival at 7 days under challenge was measured in the post-challenge period and every 12 h. To further assess tissue involvement, samples of the hearts, livers, spleens, lungs, and kidneys were collected from immunized and control mice at 15 h post-challenge. At the end of the study, all animals were euthanized.

### 2.8. Measurement of OMV-Specific Serum Antibody Levels

The specific IgG in serum against *P. multocida* OMVs was detected with the help of an indirect enzyme-linked immunosorbent assay (ELISA). In brief, 96-well plates were coated with purified OMVs (10 µg/mL, 100 µL/well), diluted in carbonate bicarbonate buffer (pH 9.6), and incubated overnight at 4 °C. Plates were washed using PBST (PBS with 0.05% Tween-20) and blocked with 5% BSA in PBST for 1 h at room temperature. Essentially, the serum samples were diluted 1:100 in PBST with 1% BSA added to the plates in duplicate and incubated at room temperature over 2 h. Secondary antibodies used were HRP-conjugated goat anti-mouse IgG, IgG1, and IgG2a (ABclonal, Wuhan, China),were put in after washing and incubated for 1 h. TMB substrate was used to detect bound antibodies, and the reaction ended with the addition of 1 N H_2_SO_4_. The absorbance at 450 nm was recorded using a microplate reader, and the optical density values obtained were taken as an indication of OMV-specific antibody responses.

### 2.9. Yak Immunization and Serological Evaluation

To evaluate the immunogenicity in a natural host, 21 healthy adult yaks (pre-screened as *P. multocida* seronegative) were randomly allocated into three groups (*n* = 7 per group). Adhering to the murine immunization schedule, yaks were intramuscularly administered with 500 μg OMVs, 1000 μg OMVs, or PBS on days 0 and 14. Serum samples were collected via the jugular vein on days 0, 7, 14, 21, and 28 for kinetic analysis. OMV-specific IgG levels were quantified using the indirect ELISA protocol described above for mice, with the modification of using HRP-conjugated goat anti-bovine IgG (ABclonal, Wuhan, China) as the secondary antibody. The absorbance was recorded at 450 nm to assess the induction of the humoral immune response.

### 2.10. Statistical Analysis

Statistical analysis and graph plotting were performed using GraphPad Prism 9.5 software. Data are presented as mean ± standard error of the mean (SEM). Differences between two groups were analyzed using the *t*-test, while differences among multiple groups were analyzed using One-way ANOVA followed by Tukey’s multiple comparison test. A *p*-value less than 0.05 was considered statistically significant (* *p* < 0.05), highly significant (** *p* < 0.01), extremely significant (*** *p* < 0.001), and very highly significant (**** *p* < 0.0001).

## 3. Results

### 3.1. Characterization of OMVs Derived from P. multocida

**Figure 1:**
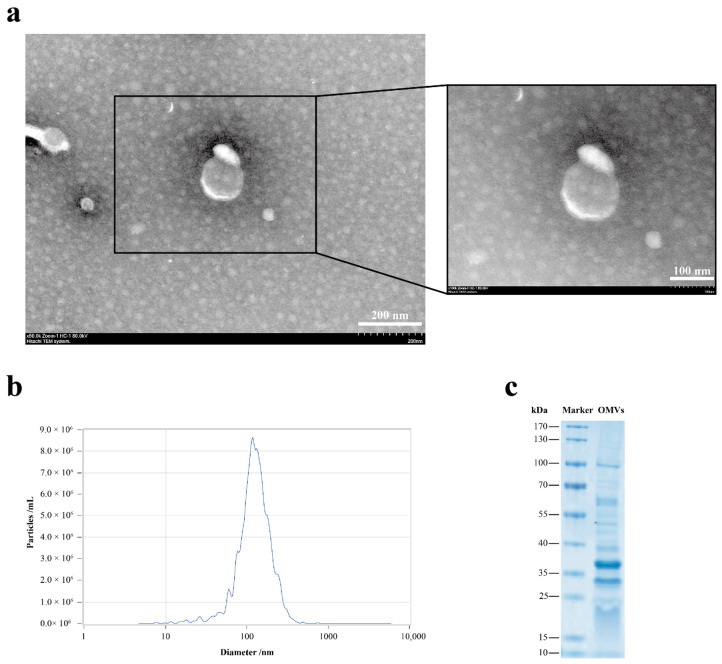
Morphology and particle size of OMVs of *P. multocida*. (**a**) Transmission electron microscopy of the isolated OMVs of *P. multocida* (200 nm, 100 nm). (**b**) Nanoparticle-tracking of OMVs. (**c**) The preparation of SDS-PAGE of whole protein of isolated *P. multocida* OMVs using a protein ladder of 170 kDa.

(Figure 1a) illustrates that the isolated vesicles had a typical cup-shaped or spherical appearance that contained a clear bilayer membrane, which is synonymous with the classical ultrastructural features of the bacterial OMVs that had previously been described.

NTA was also done to ascertain the size distribution and the concentration of purified OMVs. The analysis demonstrated the concentration of 2.8 × 10^7^ particles/mL in the dilute sample. The dilution factor was set at 4000, which was used to calculate the original OMV preparation concentration in the approximate range of 1.1 × 10^11^ particles/mL. The particle size distribution was between about 20 and 300 nm with two major peaks at 102.7 and 146.5 nm, respectively, and a median particle diameter of 103.9 nm (Figure 1b). The size range of membrane vesicles observed here is usually in agreement with the previous reports [20]. Furthermore, SDS-PAGE analysis of the purified OMVs revealed multiple protein bands, most of which were distributed between 25 and 100 kDa, with two prominent bands detected at approximately 35 kDa (Figure 1c). These findings indicate that the OMVs contain a diverse array of vesicle-associated proteins.

### 3.2. P. multocida OMVs Potently Induce Inflammatory Gene Expression in RAW264.7 Macrophages

**Figure 2:**
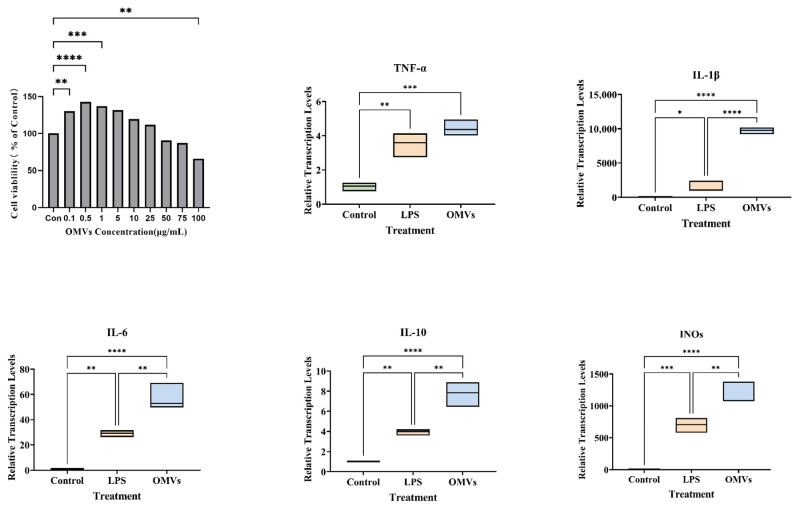
OMVs induce innate immune-associated inflammatory responses at the transcriptional level. Relative mRNA expression levels of TNF-α, IL-1β, IL-6, iNOS, and IL-10 in RAW264.7 macrophages following stimulation with PBS, LPS, or OMVs were determined by qRT-PCR. Data are presented as mean ± SD after normalization to the internal reference gene. Statistical significance was analyzed by one-way ANOVA. * *p* < 0.05, ** *p* < 0.01, *** *p* < 0.001, **** *p* < 0.0001.

To determine whether *P. multocida* OMVs activate innate immune responses in macrophages, the expression of inflammation-related genes was evaluated in RAW264.7 cells after stimulation with PBS, LPS, or OMVs (Figure 2). LPS and OMVs both dramatically enhanced the expression of TNF-α, IL-1β, IL-6, iNOS, and IL-10 relative to controls treated with PBS. Notably, OMVs caused a stronger reaction than LPS, specifically in the case of TNF-α, IL-1β and IL-6, which were all greatly expressed in cells stimulated with OMVs. Of these, IL-1β was the most activated. iNOS expression, which is indicative of activation of inflammatory effector mechanisms, was also significantly enhanced by the OMV treatment. In parallel, IL-10 transcription was also markedly upregulated, suggesting that OMVs induce not only a robust pro-inflammatory response but also a concomitant regulatory response. Collectively, these findings indicate that *P. multocida* OMVs are potent activators of macrophage inflammatory responses in vivo at the transcriptional level.

### 3.3. P. multocida OMVs Undergo Time-Dependent Uptake by RAW264.7 Macrophages

**Figure 3:**
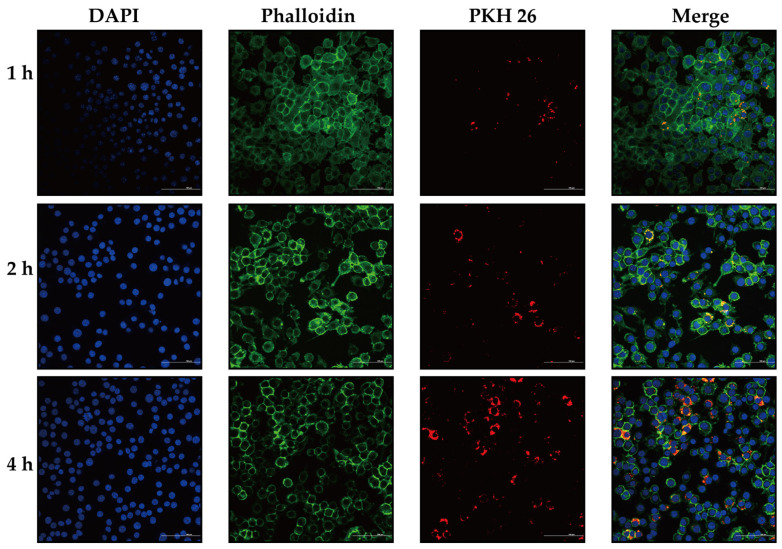
Time-dependent uptake of OMVs by RAW264.7 macrophages. RAW264.7 cells were incubated with PKH26-labeled OMVs for 1, 2, and 4 h and analyzed by confocal laser scanning microscopy. Nuclei were stained with DAPI (blue), the cell membrane was labeled green by phalloides, and OMVs were labeled with PKH26 (red). Representative merged images show progressive intracellular accumulation of OMVs in RAW264.7 cells over time. Representative images from three independent experiments are shown. Scale bar = 100 μm.

In order to find out whether *P. multocida* OMVs are internalized by macrophages, PKH26-labeled OMVs were introduced into RAW264.7 cells and observed under the confocal microscope at the given time points (Figure 3). The appearance of red fluorescent signals in the cytoplasmic region as early as 1 h post-incubation indicated rapid association of OMVs with macrophages, followed by their internalization. The level of intracellular fluorescence signal also started to rise gradually at 2 h and was significantly elevated at 4 h, which correlates with accumulation of the vesicles in the cell with time. The nuclear morphology was not affected in the course of the experiment, which is shown by DAPI staining. These findings collectively demonstrate that *P. multocida* OMVs are easily absorbed by RAW264.7 macrophages, indicating that they directly interact with innate immune cells [21].

### 3.4. Proteomic Characterization of P. multocida OMVs

To determine the protein cargo and elucidate the functional potential of *P. multocida* OMVs, a comprehensive proteomic analysis was performed. A total of 1213 proteins were identified by comparing the OMV proteome to the *P. multocida* reference database. Notably, several proteins critical for membrane integrity and host–pathogen interactions were identified, including outer membrane proteins (OmpH, Omp16, OmpA, and OmpW), the transferrin-binding protein TbpA, and the protective lipoprotein PlpP [21,22].

**Figure 4:**
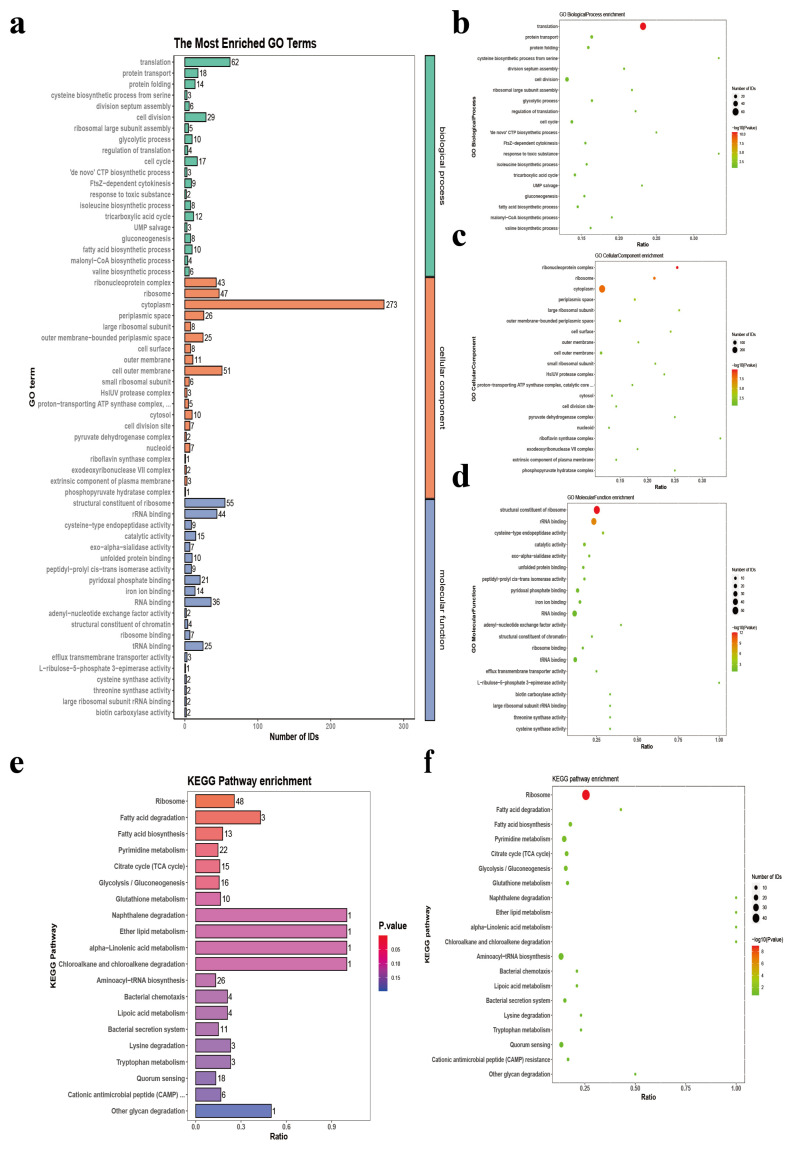
Functional annotation and enrichment analysis of *P. multocida* OMV proteome. (**a**) Distribution of top enriched GO terms across Biological Process (BP), Cellular Component (CC) with an abbreviatory term, proton-transporting ATP synthase complex, catalytic core F (1), and Molecular Function (MF) categories based on protein counts. (**b**–**d**) Bubble plots illustrating GO enrichment for BP (**b**), CC (**c**), and MF (**d**). (**e**) KEGG pathway distribution showing the proportion of identified involved in major metabolic and signaling pathways. (**f**) Bubble plot of KEGG pathway enrichment. For all bubble plots (**b**–**d**,**f**), the x-axis represents the enrichment ratio, bubble size indicates the number of proteins, and the color scale reflects statistical significance (−log_10_ adjusted *p*-value). The OMV cargo is predominantly associated with translation, ribosomal structure, core metabolism, and membrane organization.

Functional annotation of the OMV proteome was conducted using Gene Ontology (GO) enrichment analysis. As illustrated in Figure 4a–d, the top 20 significantly enriched GO terms were categorized into three main domains: Cellular Component (CC), Biological Process (BP), and Molecular Function (MF). In the CC category, “cytoplasm” was the most predominantly enriched term (273 proteins), followed by “outer membrane” and “ribosome-bound compartments.” Within the BP domain, “translation” represented the most enriched term, involving 62 proteins, while other identified biological activities included protein transport, folding, and amino acid biosynthesis. For the MF category, “structural constituent of ribosome” (55 proteins) and “efflux transmembrane transporter activity” were significantly overrepresented.

To further explore the metabolic and signaling integration of the OMV proteome, KEGG pathway enrichment analysis was performed (Figure 4e,f). The “ribosome” pathway emerged as the most significantly enriched signaling route, containing 48 mapped proteins. Furthermore, metabolic pathways related to fatty acid biosynthesis/degradation and various amino acids (tryptophan and glutathione) were notably enriched. Interestingly, pathways associated with bacterial environmental adaptation—such as chemotaxis, quorum sensing, and cationic antimicrobial peptide (CAMP) resistance—were also overrepresented. These findings suggest that OMV secretion may play a pivotal role in *P. multocida* survival and acclimatization to the host environment [23,24]. Collectively, these data indicate that yak-derived *P. multocida* OMVs possess a complex and functionally diverse protein repertoire, reflecting their multifaceted roles in bacterial physiology and pathogenesis.

### 3.5. OMVs of P. multocida Induce Robust Humoral Immune Responses

**Figure 5:**
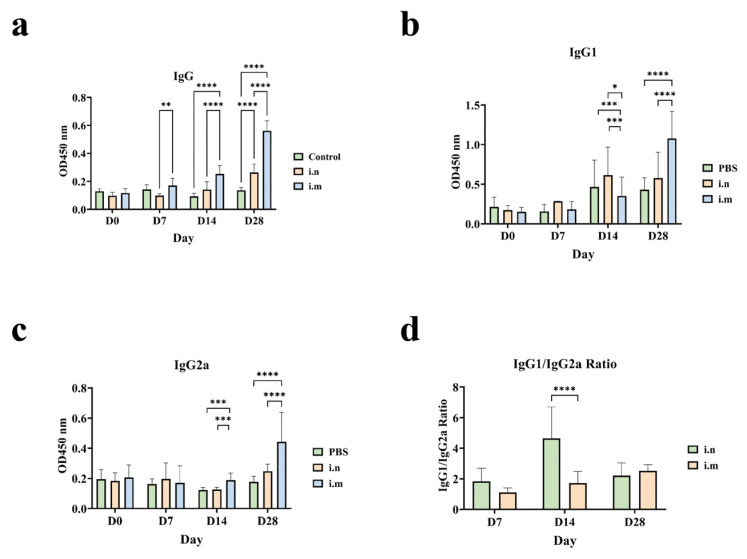
Humoral immune responses in mice immunized with *P. multocida*-derived OMVs. (**a**) The levels of serum IgG in mice more than 28 days after immunization with OMVs. (**b**) IgG1 levels of mice more than 28 days after immunization with OMVs. (**c**) The level of serum IgG2a in mice after 28 days of OMV immunization. (**d**) IgG1/IgG2a ratio of serum in mice 28 days following the administration of OMV. Data are presented as mean ± SD. Statistical significance was analyzed by one-way ANOVA. * *p* < 0.05, ** *p* < 0.01, *** *p* < 0.001, **** *p* < 0.000.

To assess the humoral immune response induced by *P. multocida* OMVs, mice were immunized intramuscularly or intranasally with purified OMVs, whereas PBS-treated mice were included as controls. Serum anti-OMVs IgG, IgG1, and IgG2a levels were determined by ELISA on days 7, 14, and 28 following primary immunization and prior to each booster dose. (Figure 5a) demonstrates that a significant rise in serum IgG levels was induced by intramuscular injection of *P. multocida* OMVs at 2 and 4 weeks after primary immunization compared to PBS (*p* < 0.0001). Serum IgG levels also increased significantly through intranasal immunization, although it was only clear at 4 weeks (*p* < 0.0001). Subclass analysis revealed that only the intramuscularly immunized group had a significant rise in IgG1 (Figure 5b) and IgG2a (Figure 5c) at 4 weeks after initial immunization in comparison with the controls (*p* < 0.0001). The IgG1/IgG2a ratio (Figure 5d) also revealed that intramuscular immunization by OMVs had preferential induction of a Th2-skewed immune response.

### 3.6. OMV Immunization Confers Protection Against Homologous P. multocida Challenge

**Figure 6:**
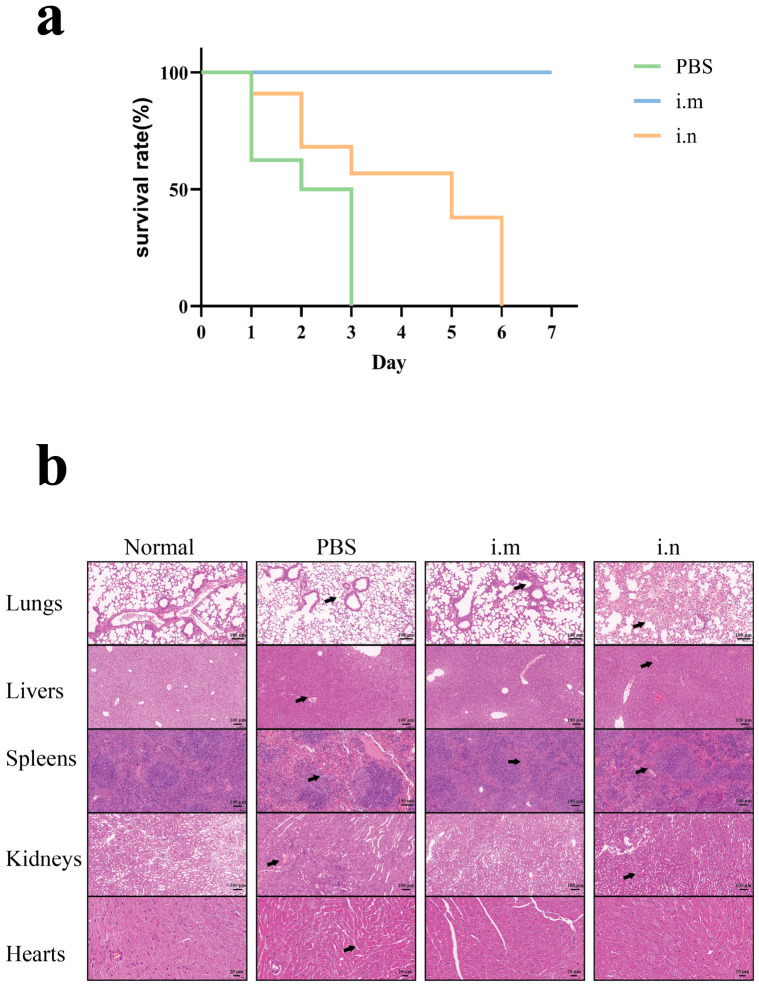
Protective efficacy of *P. multocida*-derived OMVs against homologous challenge in mice. (**a**) Survival curves of mice in each group after the 7 days where homologous *P. multocida* challenge is conducted. (**b**) Representation of lung, liver, spleen, kidney, and heart tissues of the normal non-challenged mice, PBS-treated control mice, the immunized by intramuscular injection mice and the immunized by intranasal inoculation mice by using hematoxylin and eosin (H&E) stain. The arrows demonstrate regions where there is a significant pathological change.

In order to ascertain the protective effect of *P. multocida* OMVs, a random selection of seven mice in each group was done, and they were exposed to 101 CFU of homologous strain. Figure 6a indicates that survival was followed over a 7-day period. The PBS control group did not show any survivors once subjected to the challenge, but the intramuscular administration of OMV immunization resulted in 100% protection (survival of mice). In comparison, intranasal immunization was unable to protect the mice, and there was 0% survival in this group. These findings suggest that intramuscular injection of *P. multocida* OMVs is significantly more efficient than intranasal injection in the protection of mice against homologic infection. This protective effect was further supported by the histopathological examination that was conducted 15 h after the challenge (Figure 6b). PBS-treated mice had severe hemorrhage, accumulation of erythrocytes in the alveoli and capillaries, alveolar structural disruption, and inflammatory infiltration in the lungs. The PBS and intranasal groups displayed sinusoidal congestion, erythrocyte infiltration, thickened hepatic cords, and hepatocellular degeneration and necrosis in their livers, and the intramuscular group demonstrated little pathological changes and appeared close to normal tissue morphology. Congestion and hemorrhage were observed in the spleens in both immunized groups but were more intense in intranasally immunized mice and most pronounced in PBS controls, accompanied by a loss of spleen parenchymal cells and demarcation of red and white pulp. The effects were observed in the kidneys, which included congestion, thickening of the tubular walls, luminal narrowing and shedding in the tubular epithelial cell only in all challenged groups except normal controls. The PBS-treated mice exhibited enlarged intermuscular spaces and myocardial edema in the hearts, which were reduced in animals in the vaccination groups. Altogether, these results indicate that OMV vaccination reduces tissue damage after homologous *P. multocida* challenge, and intramuscular vaccination offers better protection.

### 3.7. OMV Vaccination Induces Robust Serum IgG Responses in Yaks

**Figure 7:**
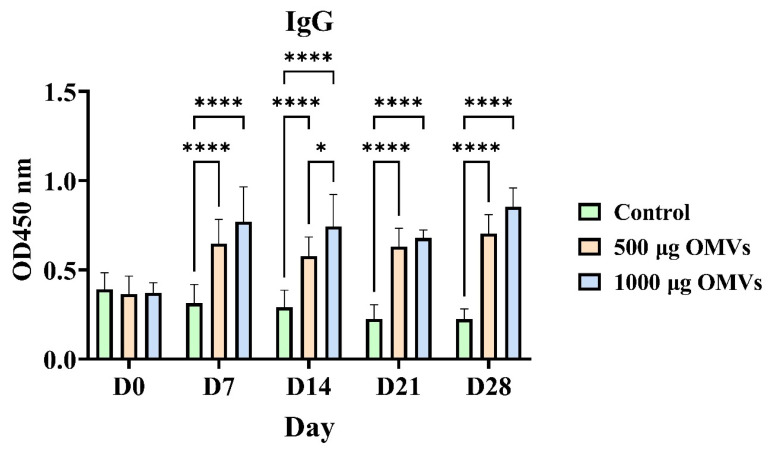
OMV vaccination elicits robust serum IgG responses in yaks. Yaks were immunized with OMVs at doses of 500 μg or 1000 μg, and serum IgG levels were measured by ELISA at D0, D7, D14, D21, and D28 post-immunization. Optical density was recorded at 450 nm (OD450). Animals in the control group received PBS. Data are presented as mean ± SD. Statistical significance was analyzed by one-way ANOVA. * *p* < 0.05, **** *p* < 0.0001.

The humoral immunogenicity of OMV vaccination was assessed by measuring serum IgG levels in yaks at various time intervals following immunization. Baseline IgG levels were not significantly different between groups at day 0 as demonstrated in (Figure 7). Conversely, yaks vaccinated with 500 μg or 1000 μg OMVs generated considerably high levels of serum IgG Antibody as compared to the control group on day 7 to day 28. In the vaccinated groups, IgG peaked at day 21 and there was a small decrease at day 28; both sets of vaccinated groups had a significantly high titer, more than the control levels. The IgG level of the 1000 μg dose persistently led to the elevation of IgG level compared to that of the 500 μg dose, which is an indication of a dose-sensitive antibody response. These findings indicate that OMV immunization presents an effective induction of potent and sustained humoral immune response in yaks.

## 4. Discussion

OMV-based vaccines developed against *Neisseria meningitidis* have been widely used worldwide to prevent epidemic meningitis in children and have demonstrated considerable effectiveness [25]. Consistent with these findings, further studies have investigated OMV-based vaccines, and numerous reports have demonstrated their potential to protect against infections caused by Gram-negative bacteria, including *Acinetobacter baumannii*, *Bordetella pertussis*, *Burkholderia pseudomallei*, *Escherichia coli*, *Klebsiella burkholderia*, *Pseudomonas aeruginosa*, and *Salmonella enterica* among others [26,27,28,29,30,31]. Moreover, previous studies have identified the protein localization profiles of OMVs secreted by two serotypes of *P. multocida* [32,33].

In this study, we identified the typical bilayer membrane structure of purified OMVs from *P. multocida* through TEM, and the results were similar with other studies on *P. multocida* [34,35]. In the meantime, we used NTA to measure OMVs. The mean size of *P. multocida* OMVs that were observed using NTA was 103.9 nm in diameter, which was lower in comparison to another study [35]. NTA, however, has shortcomings in the determination of OMV because NTA determines particles in a solution, and it is necessary to clean up the OMV prior to detection [36]. Also, the sizes of OMVs varied by different purify processions and isolation methods [36,37].

Furthermore, proteomic analysis of *P. multocida* OMVs was conducted to explore their potential functional relevance. GO enrichment and KEGG pathway analyses were applied to characterize the major proteins identified in the vesicles. GO enrichment analysis showed that a considerable proportion of these proteins were cytoplasmic in origin, which may be associated with the biogenesis and functional roles of OMVs. Previous research has indicated that phage-mediated explosive cell lysis may be a ubiquitous mechanism responsible for the generation of membrane vesicles in Gram-negative bacteria and for the release of cytosolic public goods in bacterial biofilms [38]. Meanwhile, a substantial proportion of the identified proteins were found to be associated with ribosomes. According to a recent review, RNA is a typical cytosolic component that is present in cytoplasmic membrane vesicles (CMVs) and E-type MVs of Gram-negative bacteria but is expected to be absent from OMVs; it also remains to be elucidated whether it has a role in MV-mediated interbacterial or interkingdom interaction [39]. Consistent with the consensus that OMPs are the major protein components of bacterial OMVs, we identified multiple well-characterized immunogenic OMPs in our samples, including OmpA, OmpH, Omp16, OmpW, TbpA, and PlpP. Based on our proteomic data and the previous literature, OmpA and OmpH appear to be the most potent antigenic candidates within the *P. multocida* OMV repertoire. OmpA, identified with the highest number of PSMs, is highly conserved and essential for bacterial structural integrity and host cell adhesion [40]. Similarly, OmpH is a well-recognized protective antigen capable of inducing robust cross-protection [41]. The high abundance and inherent immunogenicity of these OMPs likely render them the primary drivers of the OMV-mediated immune response. An intriguing observation in our study was that OMV immunization induced higher levels of pro-inflammatory cytokines (TNF-α, IL-1β, IL-6) compared to purified LPS. This enhanced inflammatory response may be attributed to the complex composition of OMVs, which act as multi-component “PAMP-shuttles”. Unlike purified LPS which primarily triggers the TLR4 pathway, OMVs carry a diverse array of pathogen-associated molecular patterns (PAMPs), including lipoproteins (TLR2 ligands), peptidoglycans (NOD1/2 ligands), and potentially bacterial DNA/RNA (TLR7/9 ligands) [42]. The simultaneous activation of multiple pattern recognition receptors (PRRs) likely leads to a synergistic signaling effect, resulting in a more potent innate immune activation than LPS alone [43]. These characteristics underscore the potential of OMVs to serve as self-adjuvanting vaccine platforms, although the balance between immunogenicity and systemic toxicity requires careful calibration for clinical use.

The OMV has been widely researched as a candidate vaccine in recent years, given that it contains a variety of antigens and can be efficiently recognized and taken up by antigen-presenting cells. In this study, there was a marked increase in serum IgG level at the 28 days post-immunization from both intramuscular and intranasal mice. Moreover, by comparing tissue sections with control mice, we found that intramuscular *P. multocida* OMVs might significantly reduce hemorrhage and cellular damage in the lung and liver. In another study, the researcher found that vaccination with *P. multocida* OMVs could significantly reduce the nasopharyngeal colonization rates of *P. multocida* [44]. However, the serum IgA levels of intranasal mice were not significantly increased, which might be interrelated with the ways of vaccination [43].

Regarding protective efficacy, we observed differences in survival rates between intranasal (IN) and intramuscular (IM) vaccination groups. It is important to note that while IN vaccination is designed to stimulate mucosal immunity to block *P. multocida* at its natural point of entry, the injectable challenge used in this study bypasses this primary mucosal barrier. This discrepancy may explain why the IM route, which excels at inducing high-titer systemic IgG, often yields superior survival results in experimental models involving systemic lethal challenges [45]. Future studies utilizing the intranasal challenge model would be more representative of natural bovine hemorrhagic septicemia and might better highlight the unique advantages of IN-induced mucosal protection.

Most importantly, we extended our evaluation from the mouse model to the natural host and demonstrated that OMV immunization induced robust and dose-dependent humoral immune responses in yaks. This is a critical step towards clinical application, as immune responses in mice often do not accurately predict vaccine efficacy in large animals due to significant differences in immune system composition and pathogen–host interactions. Our results provide the first experimental evidence that *P. multocida* OMVs are safe and immunogenic in yaks, laying a solid foundation for further field trials and vaccine development.

## 5. Conclusions

This study provides compelling evidence that yak-derived *P. multocida* OMVs are biologically active and highly immunogenic nanostructures with significant vaccine potential. Proteomic profiling showed that OMVs possess a universal array of proteins of ribosomal activity, membrane organization, metabolism, and bacterial adaptation, indicating their diverse biological significance. Immunization experiments also revealed that OMVs were efficient in inducing strong humoral immunity in mice and yaks with intramuscular inoculation, causing the best antibody response and a Th2-biased immune response in mice. It is important to note that intramuscular OMV vaccination was able to provide full protection against homologous challenge, as well as significantly decrease tissue damage to infected organs. Collectively, the results make *P. multocida* OMVs promising in terms of OMV-based vaccine development and provide useful experimental support in future translational studies to control *P. multocida* infections in livestock.

## Data Availability

Data is contained within the article.
